# Editorial: Synaptic Failure and Circuits' Impairment—Cognitive and Neurological Disorders—Moving a Step Forward

**DOI:** 10.3389/fnmol.2022.979511

**Published:** 2022-07-13

**Authors:** Touqeer Ahmed, Jaewon Ko

**Affiliations:** ^1^Neurobiology Laboratory, Department of Healthcare Biotechnology, Atta-ur-Rahman School of Applied Biosciences, National University of Sciences and Technology, Islamabad, Pakistan; ^2^Department of Brain Sciences, Daegu Gyeongbuk Institute of Science and Technology (DGIST), Daegu, South Korea

**Keywords:** synapses, neural circuits, synaptic dysfunction, neurological disorders, cognitive functions

Synapses act as the core information processing unit in the brain and are fundamental for various cognitive processes during development, adulthood, and aging. Thus, elucidation of how synapses are constructed, maintained, remodeled, and eliminated is essential for better understanding how various neurological and/or cognitive disorders occur. An area of active investigation is how dysfunctions of key synaptic signaling mechanisms contribute to diseased conditions.

The present Research Topic presents a series of observations that could provide new perspectives on the molecular, cellular, and systems mechanisms implicated in various neurological and cognitive disorders. Badawi et al. studied the role of salt-inducible kinases (SIKs), which were previously implicated in infantile epileptic encephalopathy and autism spectrum disorders (ASDs). The authors used SIK1 mutant mice that harbor mutations observed in patients with ASDs and exhibit disruption of excitatory and inhibitory synaptic balance and ASD-like behaviors, and found that risperidone administration significantly improved their repetitive behaviors and decreased the levels of neuronal excitability and excitatory synaptic transmission. Synaptic cell-adhesion proteins have been considered prime candidates for various neurological and neuropsychiatric disorders. Uchigashima et al. extensively discuss the physiological and pathological roles of neuroligin-3, which has been strongly associated with non-syndromic ASDs and other neurodevelopmental conditions. Meanwhile, Maxeiner et al. performed sophisticated analyses using sequences from a male gerbil genome to reveal a new perspective on the evolution of neuroligin-4, which has been strongly linked to ASDs. NMDA receptors are critical for proper brain physiology and have been associated with the pathophysiology of multiple brain diseases. SLC6A20A is a proline and glycine transporter known to regulate glycine homeostasis and NMDA receptor function. Kim et al. generated heterozygous and homozygous mutant mice lacking *Slca6a20a* and performed extensive behavioral analyses. The authors found that heterozygous and homozygous Slc6a20a deletions in mice lead to differential changes in behaviors and transcriptomic profiles that could be relevant to ASD pathogenesis.

Circadian disruptions have long been suspected to play a role in the vulnerability to a subset of neurological disorders. Jiang et al. provide intriguing evidence that the activity of Rac1, a key signaling regulator involved in synapse development and plasticity, undergoes time of day-dependent alterations and mediates contextual fear memory in rats. Andrade-Talavera et al. reviewed the literature showing that spike timing dependent plasticity (STDP) and neuronal circuit rhythms are disrupted in Alzheimer's disease (AD). The authors highlight that the accumulation of neurotoxic peptides, activation of microglial cells, and impaired functions of astrocytes contribute to spike-timing precision deterioration and neuronal network collapse, leading to cognitive impairment. This review also elaborates the shortcomings of available animal models for AD and proposes the need for more studies focusing on the earlier phases of AD.

Ueda et al. studied and compared the important brain areas involved in emotional states, such as the central nuclei of the amygdala (CeL) and the bed nucleus of the stria terminalis (BNST). Some previous studies revealed many similarities in these nuclei, while others highlighted differences between them. This study elaborates that CeL and BNST neurons exhibit similar genetic compositions and gene expression profiles, but different gene expression responses to fearful stimuli. Moreover, manipulation of these nuclei has differential effects on behavior: inhibiting CeL PKCδ^+^ neurons attenuates fear learning, while inhibiting BNST PKCδ^+^ neurons affects anxiety-like behavior. Skirzewski et al. provide insightful perspectives on the significance of multisensory integration to sensory perception in brain functions, focusing on the role of neural circuits encompassing the medial prefrontal cortex and basolateral amygdala in visual information processing. The authors propose potentially promising therapeutic strategies of manipulating mPFC signaling and employing environmental enrichment to address higher-order visual dysfunctions, such as cerebral visual impairment.

Continuing efforts to elucidate culprit genes, signaling pathways, neurons, and neural circuits will provide new insights into how the brain operates at the synapse and circuit levels while engaging in higher cognitive functions. The collection of articles presented in this Research Topic cover important aspects of synaptic modulation in learning, memory, and higher cognitive functions. The above-mentioned studies demonstrate advancements in understanding synapses and circuits in the brain.

## Author contributions

Both authors listed have made a substantial, direct, and intellectual contribution to the work and approved it for publication.

## Funding

This study was supported by the National Creative Research Initiative Program (NRF-2022R1A3B1077206 to JK) and HEC NRPU-9780 grant awarded to TA.

## Conflict of interest

The authors declare that the research was conducted in the absence of any commercial or financial relationships that could be construed as a potential conflict of interest.

## Publisher's note

All claims expressed in this article are solely those of the authors and do not necessarily represent those of their affiliated organizations, or those of the publisher, the editors and the reviewers. Any product that may be evaluated in this article, or claim that may be made by its manufacturer, is not guaranteed or endorsed by the publisher.

